# A decentralized, prospective, observational study to collect real-world data from patients with myasthenia gravis using smartphones

**DOI:** 10.3389/fneur.2023.1144183

**Published:** 2023-08-01

**Authors:** Sandra Steyaert, Meelis Lootus, Chethan Sarabu, Zeenia Framroze, Harriet Dickinson, Emily Lewis, Jean-Christophe Steels, Francesca Rinaldo

**Affiliations:** ^1^Sharecare, Inc., Atlanta, GA, United States; ^2^Stanford University, Center for Bioinformatics Research, Palo Alto, Santa Clara, CA, United States; ^3^Global Real World Evidence, UCB Slough, Slough, United Kingdom; ^4^UCB S.A. (Headquarters) Allée de la Recherche, Brussels, Belgium

**Keywords:** myasthenia gravis, observational study, decentralized, smartphone-based data collection, exacerbations

## Abstract

**Introduction:**

We conducted a 3-month, prospective study in a population of patients with Myasthenia Gravis (MG), utilizing a fully decentralized approach for recruitment and monitoring (ClinicalTrials.gov Identifier: NCT04590716). The study objectives were to assess the feasibility of collecting real-world data through a smartphone-based research platform, in order to characterize symptom involvement during MG exacerbations.

**Methods:**

Primary data collection included daily electronically recorded patient-reported outcomes (ePROs) on the presence of MG symptoms, the level of symptom severity (using the MG-Activities of Daily Living assessment, MG-ADL), and exacerbation status. Participants were also given the option to contribute data on their physical activity levels from their own wearable devices.

**Results:**

The study enrolled and onboarded 113 participants across 37 US states, and 73% (N= 82) completed the study. The mean age of participants was 53.6 years, 60% were female. Participants were representative of a moderate to severe MG phenotype, with frequent exacerbations, high symptom burden and multiple comorbidities. 55% of participants (N=45) reported MG exacerbations during the study, with an average of 6.3 exacerbation days per participant. Median average MG-ADL scores for participants during self-reported exacerbation and non-exacerbation periods were 7 (interquartile range 4-9, range 1-19) and 0.3 (interquartile range 0-0.8, range 0-9), respectively. Analyses examining relationships between patient-reported and patient-generated health data streams and exacerbation status demonstrated concordance between self-reported MG-ADL scores and exacerbation status, and identified features that may be used to understand and predict the onset of MG symptom exacerbations, including: 1.) dynamic changes in day-to-day symptom reporting and severity 2.) daily step counts as a measure of physical activity and 3.) clinical characteristics of the patient, including the amount of time since their initial diagnosis and their active medications related to MG treatment. Finally, application of unsupervised machine learning methods identified unique clusters of exacerbation subtypes, each with their own specific representation of symptoms and symptom severity.

**Conclusion:**

While these symptom signatures require further study and validation, our results suggest that digital phenotyping, characterized by increased multidimensionality and frequency of the data collection, holds promise for furthering our understanding of clinically significant exacerbations and reimagining the approach to treating MG as a heterogeneous condition.

## Introduction

Generalized myasthenia gravis (MG) is a rare, chronic, heterogeneous, and unpredictable autoimmune disease that is characterized by muscle weakness and fatigue (1, 2). MG has a variety of clinical presentations but may affect functions related to breathing, swallowing, speech and moving parts of the body such as the arms and legs. A large proportion of MG patients (>50%) also have ocular involvement such as drooping eyelids (ptosis) and/or double vision, either at presentation or during the later course of the disease (3). MG is more common in young females (<40) and in older men (>60) (4). Symptom burden varies greatly among patients and often improves with periods of rest. Due to its propensity to affect each patient differently, MG is often referred to as a “snowflake” disease.

A myasthenic crisis is defined as severe weakness of the muscles that control breathing or speech and swallowing ability to the point where supportive feeding, intubation or ventilation is required. Approximately 15%–20% of people with MG experience at least one myasthenic crisis in their lives (5, 6). Any acute increase in symptom severity (also known as an exacerbation in MG symptoms), could be regarded as a possible precursor stage of a crisis and requires rapid monitoring and careful treatment. Timely clinical intervention and medication use can help relieve severe symptoms and help to avoid repetitive cycles of exacerbations and crises (7). Depending on the specific symptoms and respective severities, several therapies are available to help relieve symptom burden, such as thymectomy, anti-cholinesterase medications, immunosuppressants, steroids, and monoclonal antibody treatment (8, 9). It can be challenging to study the presence of symptom exacerbations and MG crisis, due to the low prevalence of the condition, and the variety of settings and specialties in which patients are seen and treated (leading to infrequent, random and thereby fragmented care records and traceability). The use of real-world data and digital phenotyping offer a promising solution to these challenges, enabling researchers to collect patient-generated health data (PGHD) that captures the unmet needs of patients. In particular, the ubiquity of smartphones and wearable devices allows data such as symptom occurrence and exacerbations to be collected more frequently and passively as compared to traditional, site-based clinical studies, creating a more complete picture of the lived experience of the disease. These technologies also facilitate the collection of data in a decentralized manner, making participation in research much more accessible and convenient for patients (10).

Between October 2020 and July 2021, UCB Pharma and Sharecare conducted a 3 month prospective observational study in US adults with MG using fully decentralized methods (ClinicalTrials.gov Identifier: NCT04590716). The objectives of this study were to (i) collect self-reported outcomes and passively generated health data from MG patients covering exacerbation and non-exacerbation periods, (ii) determine concordance between self-reported exacerbation status and MG-ADL scores, (iii) characterize symptom involvement during exacerbations, and (iv) identify potential subtypes of exacerbations. Participants in the study contributed both self-reported outcomes data as well as passively generated health data from their smartphones and wearable devices. Together with a patient’s clinical profile and history, this multidimensional PGHD could prove to be invaluable in identifying subgroups of MG patients based on symptom clusters that will help with therapeutic decisions and prognosis, and thus overall better individualized care (11).

## Materials and methods

### Study design and data collection

This was a 3 month, prospective, observational cohort study conducted between October 2020 and July 2021 with no on-site monitoring (ClinicalTrials.gov Identifier: NCT04590716). The 3 month observation period was selected to capture an adequate number of symptom exacerbations during the study period, given a target enrollment of 100–200 participants (see Supplementary Table S1). The study was designed and conducted using a specialized platform for decentralized research that allows for multidimensional data collection directly from participants’ smartphones. All data were recorded directly by participants on their personal internet-enabled smartphones using the study mobile app and were considered source data. Data from enrolled participants were uploaded from their device, centrally stored on HIPAA-compliant cloud provider (Google Cloud Platform) and shared securely with data analysis servers.

This study was reviewed and approved by Salus IRB (www.salusirb.com, protocol number DOC-005-2020). The reporting of this study conforms to the STROBE statement (12).

### Participant recruitment and eligibility criteria

Participants were recruited in a fully decentralized manner from across the United States. Advertisements on social media channels directed interested individuals to a web-based landing page where they could review the research objectives, the estimated weekly time commitment to complete study tasks, and study compensation. From the landing page, potential participants had the option to continue to an internet-based screening tool where they could self-attest to statements reflecting the inclusion criteria for the study. Inclusion criteria were a documented diagnosis of MG with ocular or bulbar symptoms; age ≥ 18 years; having access to an internet-enabled smartphone capable of supporting the research app; understanding English; and being a legal resident of the US. All participants who completed the pre-screening process were reviewed by the study principal investigator. Approved participants were sent a standard email inviting them to enroll by downloading the study mobile app (available on iOS and Android operating systems) and completing a CFR part 11 compliant eConsent and signature process. Participants who completed the study and achieved ≥60% adherence to study tasks received a $250 Amazon Gift card as compensation.

### Study procedures and outcomes of interest

**Figure 1:**
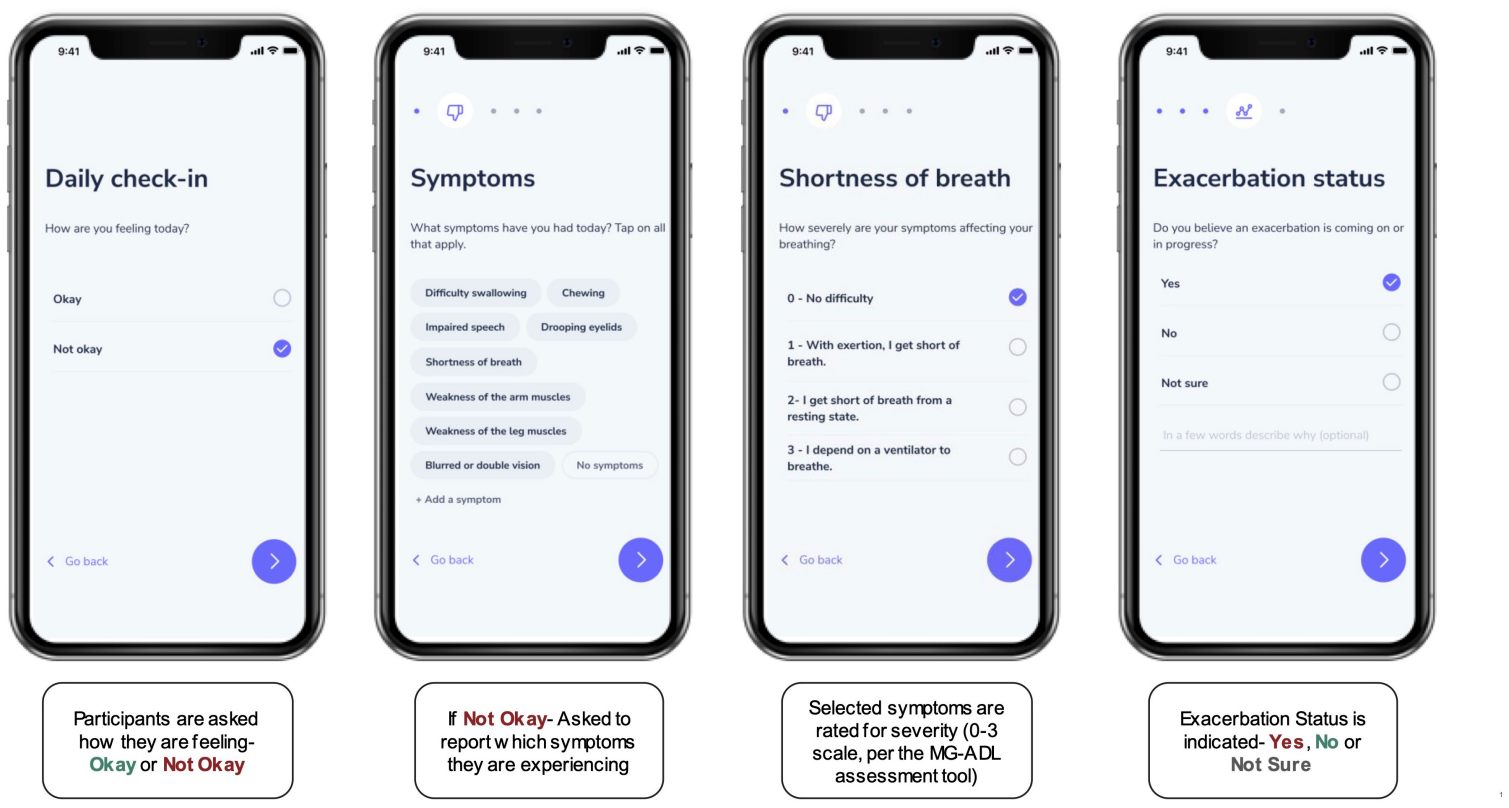
The “Daily Check-In” as a digital, self-reported outcome measure. Participants were asked to complete a Daily Check-in using the research application for the study, to self-report on symptoms and symptom severity (on a scale from 0–3) using a digital version of the MG-ADL assessment. In addition, each check-in prompted participants to indicate if they thought a symptom exacerbation was in progress by selecting “Yes,” “No,” or “Not Sure.”

Upon successful enrollment, participants were asked to confirm their MG diagnosis by uploading appropriate documentation via the study mobile app (such as a PDF of their health portal, an email or letter from their doctor confirming the diagnosis, or copies of clinical notes and laboratory results supporting the diagnosis). Participants were then invited to complete an onboarding survey to collect data on their demographic and MG disease characteristics (e.g., exacerbation frequency), comorbid conditions, and active medications. In addition to the survey, participants were asked to report baseline MG-Activities of Daily Living (MG-ADL) assessment scores on what they perceived to be “good symptom days” – (i.e., when symptom burden was low), and during “bad symptom days” (i.e., when symptom burden was high) (13). Participants also completed daily “check-ins” to report on their symptoms, symptom severity, and exacerbation status using a digital version of the MG-ADL assessment (Figure 1). When reporting on exacerbation status, they had the option to choose between “No” (not experiencing an exacerbation), “Yes” (an exacerbation is in progress), or “Not sure” (unsure if an exacerbation is in progress). Participants could also make optional connections to contribute secondary, passive data from their smartphones or wearable devices by granting permissions for the study to draw data from the appropriate application programming interfaces (APIs). Daily step count data was retrieved for the study period via Apple HealthKit (iOS users), Google Fit (Android users) or Validic (iOS or Android users). Supplementary Table S2 details the data collection instruments employed to collect the data presented in this manuscript.

Adherence to study tasks was monitored on a weekly basis and participants who were below the target threshold (60% completion of total available study tasks) were sent automated reminder emails to re-engage with the study. After 3 consecutive reminders for inactivity, participants would be flagged for review and additional outreach by the study team. Participants who were non-responders to the outreach were withdrawn from the study.

### Data preparation

Using the International Consensus Guidelines for the Management of Myasthenia Gravis as a reference (8, 9), participants were manually assigned to medication groups based on the active medications they reported for the treatment of MG. Table 1 shows how the classification of groups was determined. Similarly, participants were also classified into diagnosis groups according to the number of years since their MG diagnosis. This was calculated by subtracting the year of initial MG diagnosis from the year at the time of study enrollment. A recent diagnosis was defined as within 2 years of study enrollment, based on studies suggesting that generalized MG usually develops within 2 years of the initial diagnosis in about 50% of patients (14). Medium and long-term diagnoses were subsequently defined as 2–5 years, or >5 years from the time of study enrollment, respectively.

**Table 1:** Classification of participants into medication groups.

Group 1 (Symptomatic Therapy)	Group 2 (Pyridostigmine + Glucocorticoids)	Group 3 (Steroid-sparing Chronic Immunosuppression)	Group 4 (Treatment for Refractory Disease)
Pyridostigmine	Patients who remain significantly symptomatic on pyridostigmine, a glucocorticoid is typically added: Prednisone (first line) Methylprednisolone	Patients with insufficient response to glucocorticoids or intolerance to chronic steroid use, the following agents are typically used: Azathioprine (first line) Mycophenolate mofetil (first line) Tacrolimus Cyclosporine Methotrexate	Patients with severe, refractory MG (or in whom treatment with first line immunosuppressive therapy is limited due to toxicity) Eculizumab Rituximab Cyclophosphamide Maintenance IVIG Maintenance plasma exchange

Participants were classified according to International Consensus Guidelines for the treatment of MG, based on the active medications they reported during study onboarding.

### Data analysis and statistical methods

Data cleaning, exploratory and statistical analyses were performed in Python (v3.9) (www.python.org) and in R statistical software (v3.6) (www.r-project.org). Correlation and non-parametric testing approaches were used to examine relationships between participant-reported and participant-generated data streams. Quantitative differences between (i) exacerbation and non-exacerbation periods and/or (ii) participant subgroups were examined.

Several methods were used to investigate symptom signatures and clustering during exacerbations, including supervised and unsupervised machine learning methods. First, a chi-square test examined if there was an equal probability of occurrence of symptoms (total counts) between exacerbation and non-exacerbation days, or if there was a discrepancy between observed and expected counts. In addition, a binomial generalized linear mixed model was trained with self-reported symptoms and the associated severity scores as the input (predictor) variables, and exacerbation status (0 = non-exacerbation, 1 = exacerbation) as the binary output:


$$Y_{\mathrm{exacerbation}\;\left(0/1\right)}=\beta_{0}+\beta_{1}{}^{*}\;\mathrm{Symptom1}+\ldots +\beta_{n}{}^{*}\;\mathrm{SymptomN}$$

Beta coefficients, which for all symptoms (independent variables) were extracted and converted to odds ratios. Value of *p*s were calculated to determine if relationships between symptoms and exacerbation status were statistically significant. Finally, an unsupervised learning approach was used to examine symptom clustering. For each participant, the reported symptoms, severity and exacerbation status were transformed to a tabular format and processed through an unsupervised clustering algorithm. Two methodologies were combined; principal component analysis (PCA) (15), followed by an unsupervised clustering method, K-Means (16). While PCA aims to find a low-dimensional representation of the observed data, unsupervised clustering methods such as K-Means are focused on finding homogeneous subgroups among the observations. Using the symptom data from the exacerbation days, PCA was used to reduce the number of features (8 symptoms of MG) in our data to two. This approach (1) improves the performance of the algorithm, (2) decreases the noise associated with input features and (3) makes it easier to visualize potential clusters. Next, the newly obtained PCA scores were incorporated into the K-means clustering algorithm. The optimal number of clusters (*k* = 4) was determined using the “elbow” method applied to the Within Cluster Sum of Squares graph for the data (16). K-means clustering results were visualized in a 2D plot using the 2 PCA scores as the x and y axes. The characteristics of each cluster were examined by summarizing the symptom severity scores. An additional feature importance analysis was performed by training a Random Forest binary classifier with a one-versus-all approach to evaluate what combination of features was most prevalent/important for each unique cluster, as compared to other clusters. The results were interpreted to determine if, based on the data streams available, it was possible to differentiate between exacerbation clusters in the study population.

## Results

### Study population characteristics

**Figure 2:**
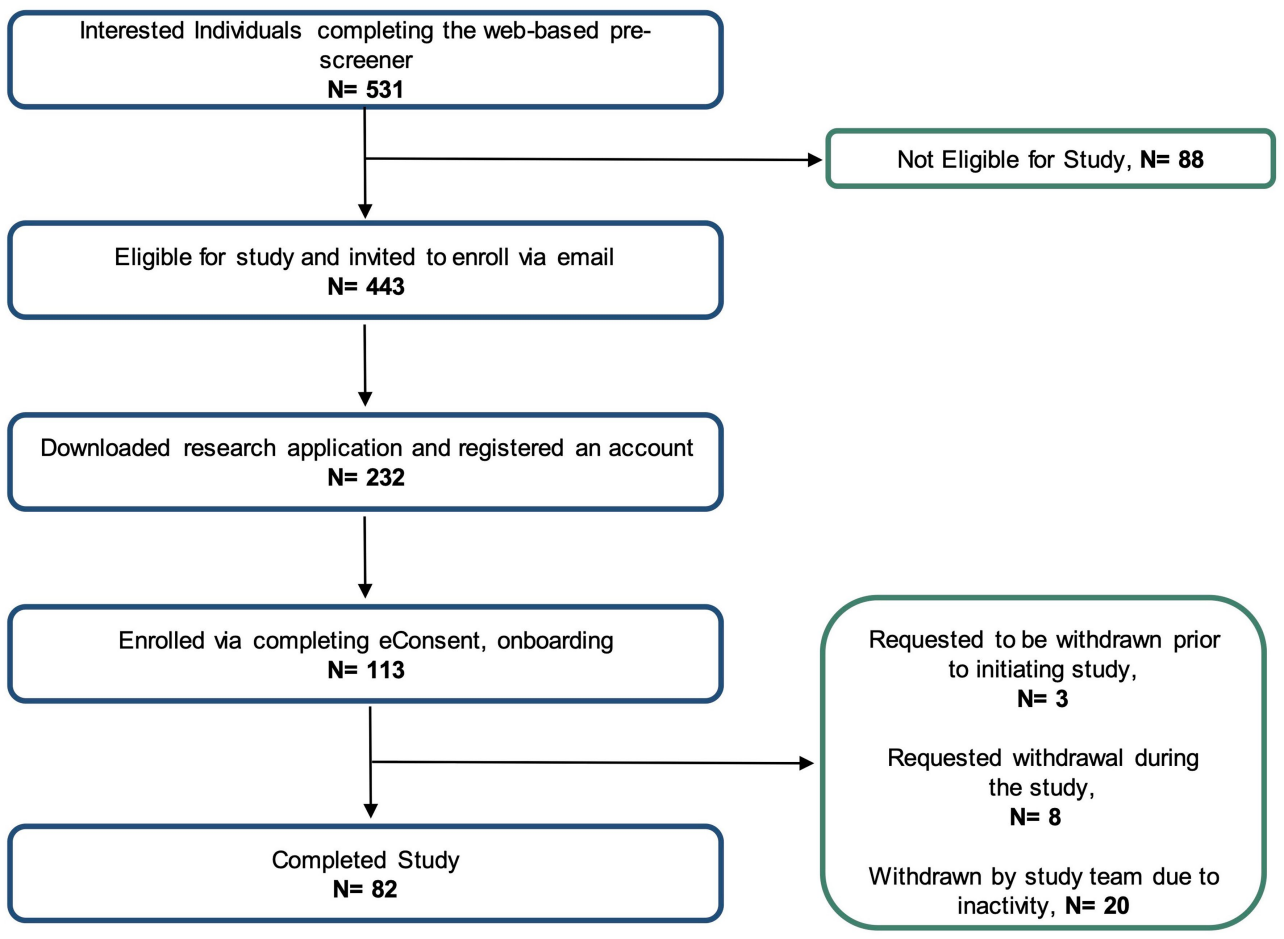
Flow diagram for enrollment at each phase of the decentralized study.

**Figure 3:**
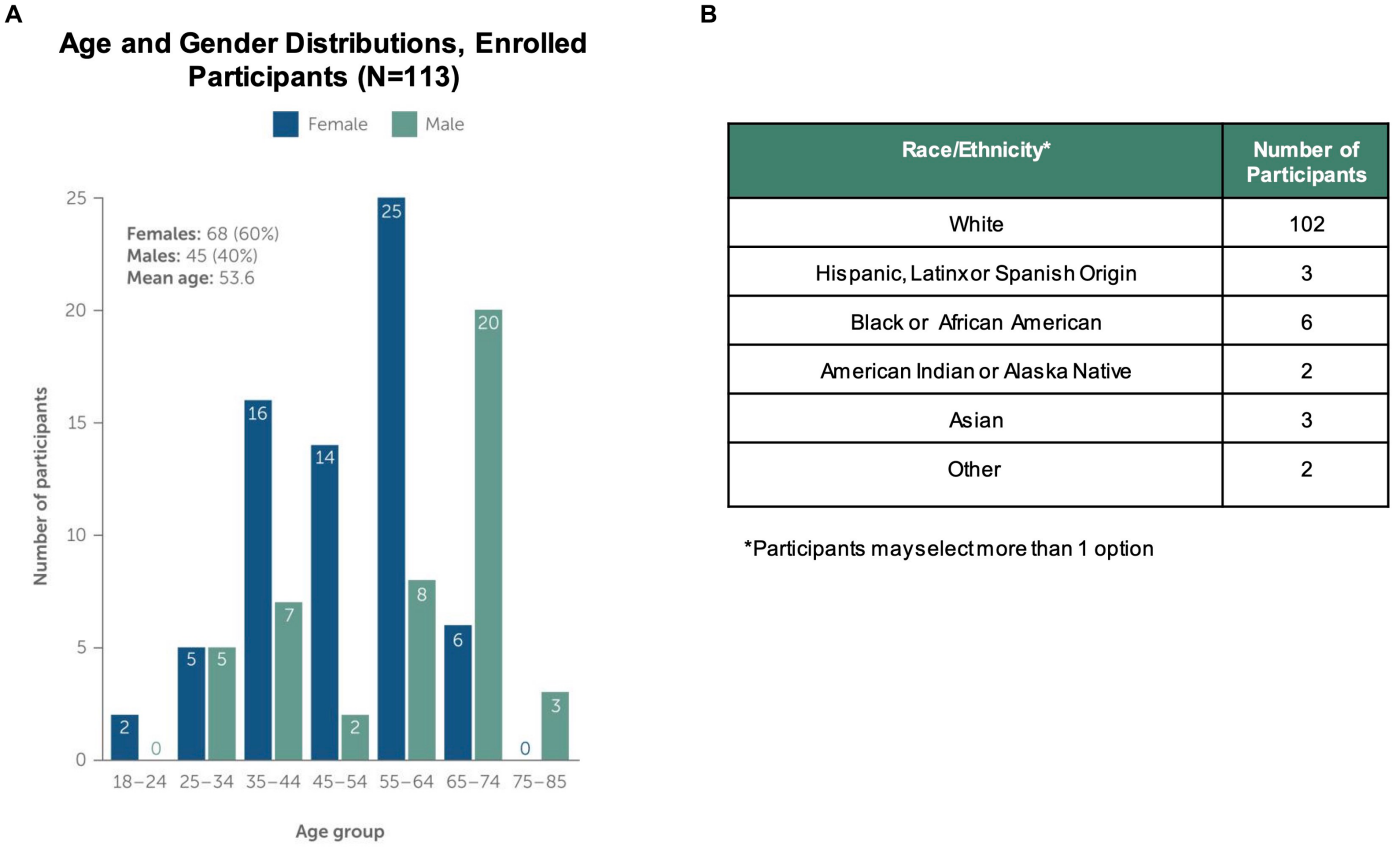
Study Population Characteristics. The study enrolled and onboarded 113 participants. The upper panels in the figure show **(A)** distribution of study participants by age, gender and **(B)** distribution of participants by self-reported Race/Ethnicity. Note that participants could select more than one race/ethnicity with which they identified.

Figure 2 shows the number of individuals at each stage of the study. Using social media ad campaigns, 531 potential participants were identified, of which 443 (83%) were approved for enrollment. 232 (52%) of approved individuals downloaded the app and registered an account. Ultimately, the study enrolled and onboarded 113 participants (25% of those identified as eligible) across 37 US states. Enrolled participants’ mean age was 53.6 years (SD 14.0), 60% were female. Enrolled participants were representative of clinically observed age and gender distributions for MG (Figure 3A) (4). Represented racial and ethnic groups were as follows: 87% White, 5% Black or African American, 3% Hispanic or Latinx, 2% Asian, 2% American Indian or Alaska Native, 2% Other (Figure 3B).

73% of enrolled participants (*N* = 82) completed the study. Participants were withdrawn for 3 primary reasons. First, a small number (*N* = 3) chose to disenroll after completing the consent and onboarding, but before initiating other study activities and data collection. Another group of participants (*N* = 8) requested withdrawal during the study course due to health or personal reasons. Finally, the majority (*N* = 20) of withdrawn participants were disenrolled due to low adherence to study tasks and non-response to outreach by the study team. For participants withdrawn due to inactivity, the average adherence to study tasks was 19.4%.

**Figure 4:**
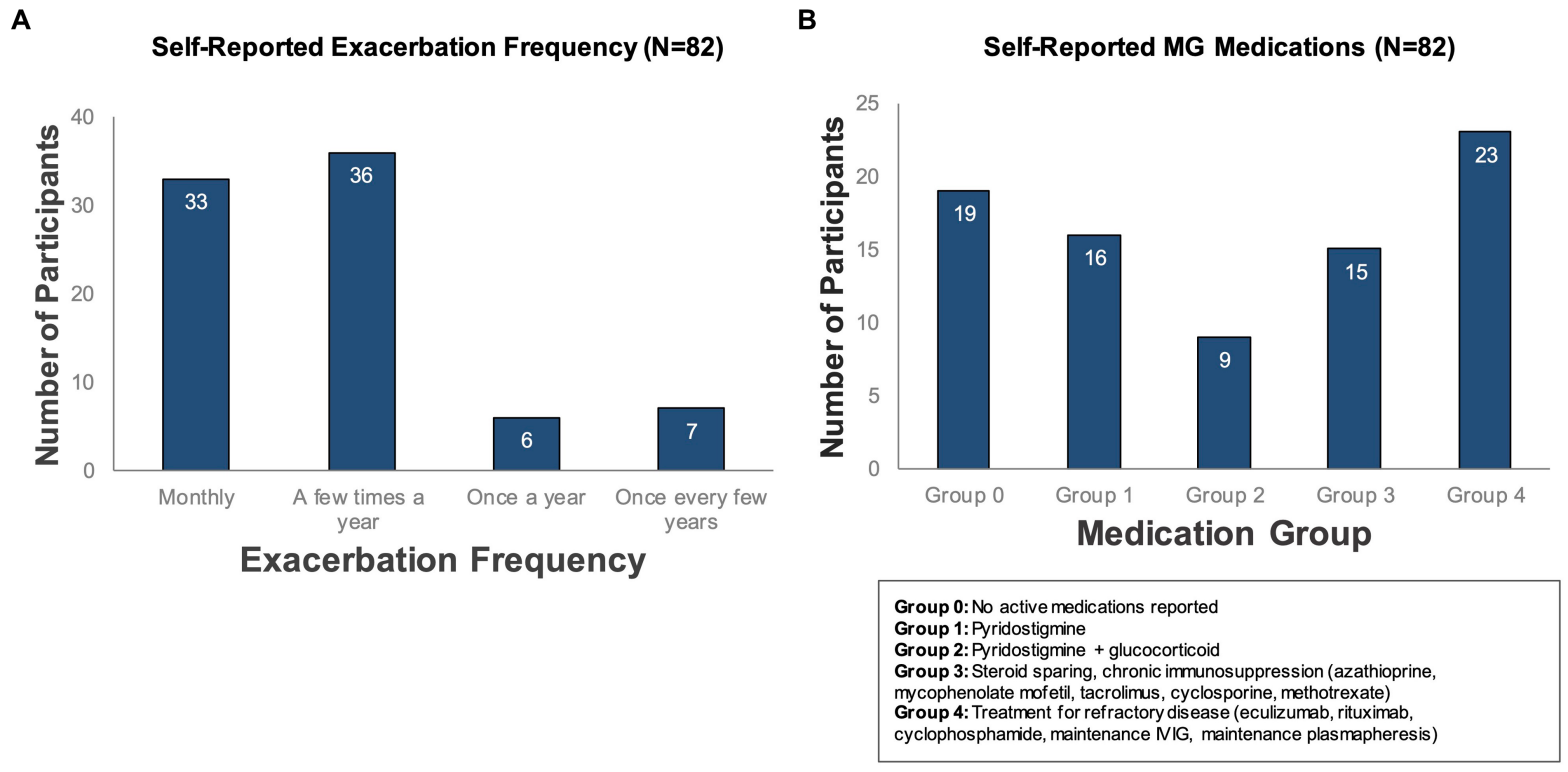
Self-reported exacerbation frequency and MG medications. Participants who completed the study (*N* = 82) were classified according to **(A)** how often they experienced symptom exacerbations (as reported in a baseline survey administered during onboarding) and **(B)** self-reported active medications for treatment of MG.

Overall, participants who completed the study (*N* = 82) represented a severe MG phenotype, based on the onboarding survey. 84% of participants reported that they experience multiple exacerbations per year (Figure 4A), with median baseline MG-ADL scores of 5 during periods of low symptom burden and 14 in periods of high symptom burden. The most frequently reported comorbid conditions were hypertension (*N* = 26), depression (*N* = 12), and type 2 diabetes (*N* = 11). In addition, the majority (63%) reported at least one active MG medication; 28% reported treatment for refractory disease. Participants were classified into pre-defined medication groups based on their self-reported active medications for treatment of MG (Figure 4B).

Table 2 summarizes the number of participants per diagnosis group, categorized according to the number of years since their initial MG diagnosis, as described in Methods (*N* = 80, 2 participants did not provide age at diagnosis during the onboarding survey). Note that 50% of participants who completed the study had a recent diagnosis of MG (within 2 years), indicating that a recent diagnosis may be a motivating factor for participating in this study and may explain the severe MG profile of our population.

**Table 2:** Classification and distribution of participants by diagnosis group.

Diagnosis Group	Group description	# Participants (*N* = 80)
Recent	Recently diagnosed (within 0–2 years of study enrollment)	40
Medium	Medium-term diagnosis (within 2–5 years of study enrollment)	14
Long	Long-term diagnosis (>5 years from time of study enrollment)	26

Participants who completed the study (*N* = 80, 2 participants did not report time of initial diagnosis) were classified into 3 groups based on the number of years between their initial MG diagnosis and study enrollment.

### Concordance between MG-ADL scores and self-reported exacerbation status

Over 4,000 data points were collected using the daily “check-ins” on MG symptoms, symptom severity and exacerbation status. 98% of the participants reported days without exacerbation (exacerbation status “No′,” total of 3,353 days), 55% reported days with exacerbations (exacerbation status “Yes,” total of 526 days) and 73% participants reported days where they were unsure if an exacerbation was ongoing (exacerbation status “Not Sure,” total of 630 days). Interestingly, in some patients, a period of “not sure” daily check-ins were reported before a self-reported exacerbation (data not shown). Participants who deteriorate less than one time a year are in a long-term stable state (*N* = 7, Figure 4A); during our study period none of these participants reported days with symptom exacerbations. Thus, we excluded these participants from the subsequent analyses, as their disease status was fundamentally different from that of the other participants.

**Figure 5:**
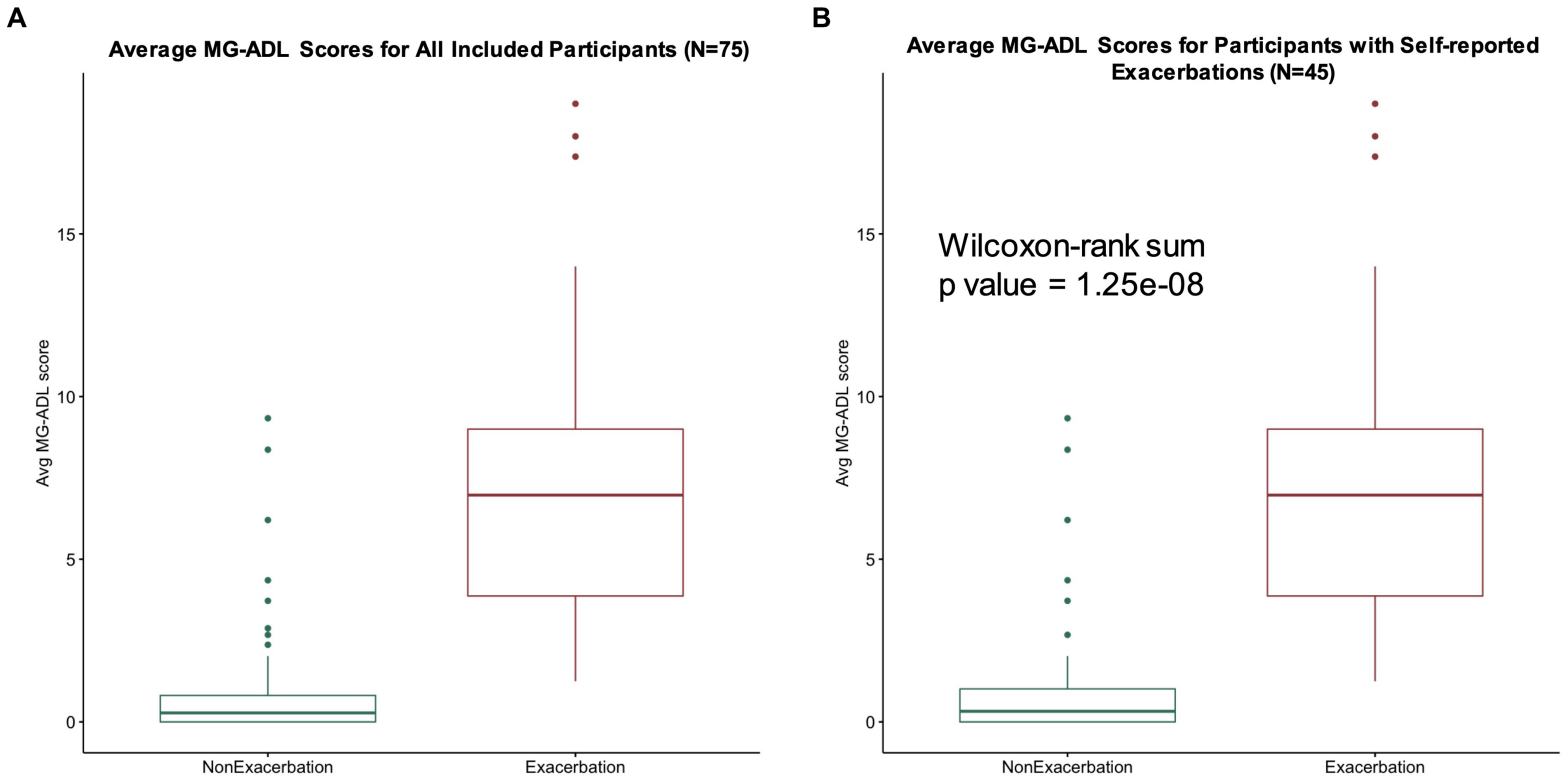
MG-ADL scores during exacerbation and non-exacerbation days. **(A)** Average MG-ADL scores per user for our study population (*N* = 75). Median average MG-ADL scores during self-reported exacerbation and non-exacerbation periods were 7 (interquartile range 4–9, range 1–19) and 0.28 (interquartile range 0–0.8, range 0–9), respectively. **(B)** Average MG-ADL scores for participants with self-reported exacerbations (*N* = 45). A significant association between average MG-ADL scores and exacerbation status was observed for this sub-cohort (Wilcoxon signed-rank value of *p* = 1.25e-08).

Median MG-ADL scores averaged per participant during self-reported exacerbation and non-exacerbation periods were 7 (interquartile range 4–9, range 1–19) and 0.3 (interquartile range 0–0.8, range 0–9), respectively for our study population of interest (*N* = 75) (Figure 5A). A total of 45 participants reported MG exacerbations, with an average of 6.3 exacerbation days per participant over the 90 day study period. A significant association between average MG-ADL scores and exacerbation status was observed for this sub-cohort (Wilcoxon signed-rank value of *p* = 1.25e-08) (Figure 5B).

### Concordance between daily step counts, MG-ADL score, and exacerbation status

**Figure 6:**
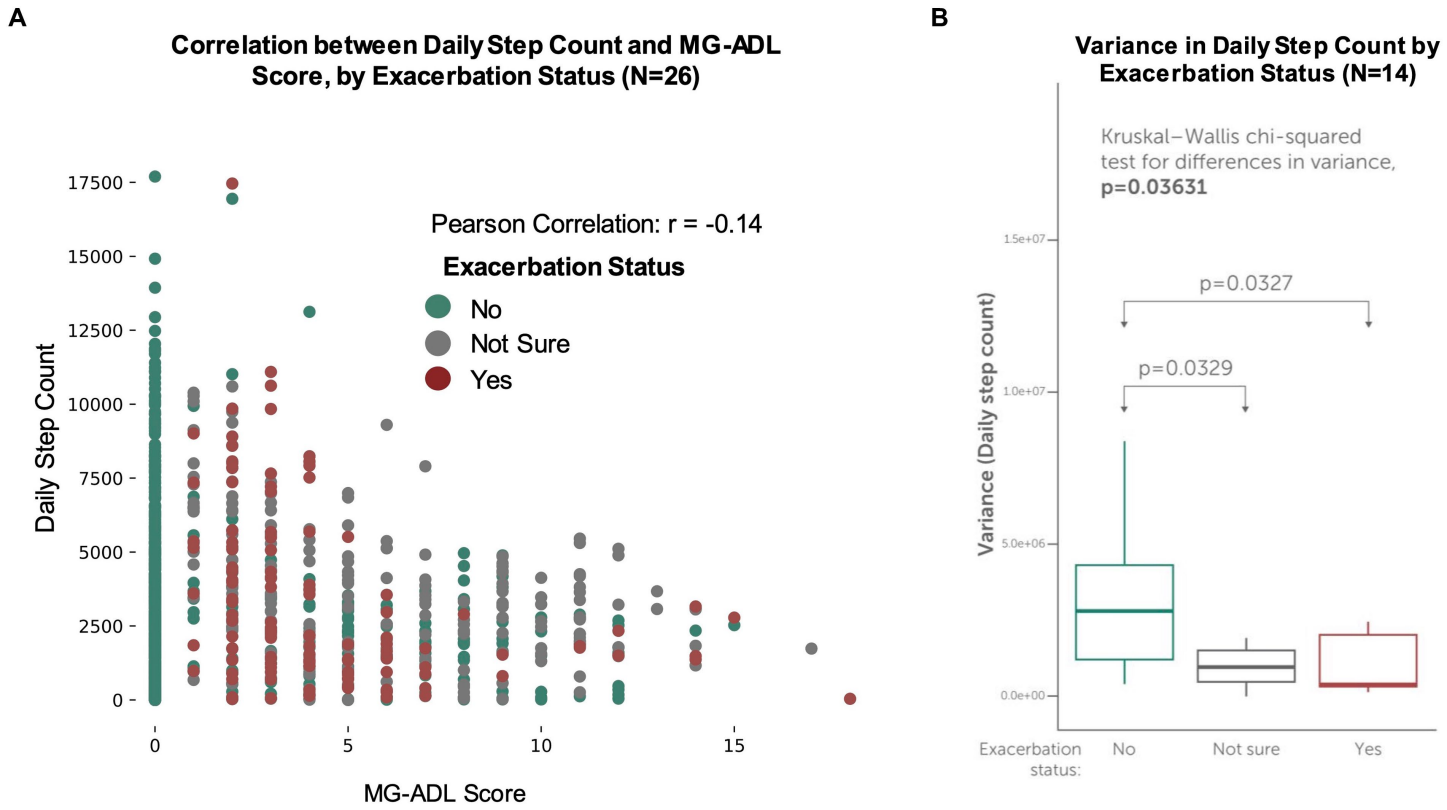
Concordance between MG-ADL scores, exacerbation status and daily step counts. Participants made optional connections to contribute secondary, passive data streams (such as their daily step count) from their smartphones or wearable devices. **(A)** Daily step counts vs. self-reported MG-ADL score for all participants with daily step counts (*N* = 26). A weak negative correlation (*r* = −0.14) was observed between daily step count and MG-ADL score. **(B)** Variance in participant-generated daily step counts for participants who experienced exacerbations (*N* = 14). For these participants, a statistically significant difference between variance in average daily step count and exacerbation status was observed (Kruskal Wallis value of *p* = 0.03), suggesting that patients who reported exacerbations took fewer steps on exacerbations days, as compared to non-exacerbation and “not sure” days.

For participants who contributed daily step count data (*N* = 26), differences in step counts during non-exacerbation and exacerbation periods were examined. Interestingly, for all participants with step counts, a weak negative correlation (Pearson correlation coefficient *r* = −0.14) between daily step count and MG-ADL score was observed (Figure 6A). For the participants who contributed daily step count data and reported exacerbations during the study (*N* = 14), a statistically significant difference between variance in average daily step count and exacerbation status was observed (Kruskal Wallis value of *p* = 0.03) (Figure 6B). This suggests that patients who reported exacerbations took fewer steps on exacerbations days, as compared to non-exacerbation days.

### Symptom representation on exacerbation and non-exacerbation days

An analysis was performed to understand whether specific symptoms were differently represented between exacerbation and non-exacerbation days for participants who reported at least one exacerbation day during the study (*N* = 45). For each symptom in the MG-ADL assessment, Table 3 summarizes the ratio of observed/expected (O/E) symptom counts for exacerbation and non-exacerbation days. A Pearson’s Chi-Squared test indicated a discrepancy between observed and expected counts for specific symptoms (value of *p* <2.2e-16).

**Table 3:** Observed/Expected (O/E) symptom ratios for exacerbation and non-exacerbation days.

MG symptom	O/E ratio, exacerbation days	O/E ratio, non-exacerbation days
Drooping eyelids	0.87	**1.25**
Shortness of breath	1.06	0.89
Difficulty chewing	1.08	0.85
Leg weakness	**0.82**	**1.34**
Arm weakness	0.88	**1.22**
Blurred or double vision	0.98	1.03
Difficulty swallowing	**1.28**	**0.46**
Impaired speech	**1.34**	**0.52**

Daily symptom data was analyzed for participants who self-reported exacerbations during the study period (*N* = 45). Cells marked in red show symptoms that were reported significantly less often than would be expected to occur by chance, whereas cells marked in blue show symptoms that were reported significantly more often than would be expected by chance.

These results suggest that in our exacerbation population, difficulty swallowing, and impaired speech were positively associated with exacerbations (i.e., these symptoms are more often reported on exacerbation days). Drooping eyelids, leg weakness and arm weakness exhibited a negative association (i.e., they are more often reported during non-exacerbation days).

To further explore the relationship between symptom combinations and exacerbation status, we trained a generalized linear mixed model with reported symptoms as the input (predictor) variables and exacerbation status (0 = non-exacerbation, 1 = exacerbation) as the binary outcome. Symptoms and symptom severity from a total of 1,717 reported daily check-ins from a subset of participants (*N* = 29) were used as input data. This subset of the data was selected from patients who reported exacerbations, who also had high level of adherence to study tasks (>60% completion), and therefore high data density. Table 4 shows value of *p*s for statistical correlation between the independent variables (participant-reported symptoms) and the dependent variable (exacerbation status). Beta coefficients for each symptom were extracted and converted to odds ratios. This analysis demonstrated that in addition to difficulty swallowing and impaired speech, shortness of breath and blurred or double vision were associated with self-reported exacerbations in this subset of participants (odds ratios of 2.67, 2.44, 1.68, and 1.39, respectively). Conversely, drooping eyelids and leg weakness were negatively associated with self-reported exacerbations (odds ratios of 0.75 and 0.67, respectively). Interestingly, the odds ratio for arm weakness was close to 1, suggesting there is no significant change in the predictor when this input feature changes.

**Table 4:** Results of a generalized linear mixed model for prediction of exacerbation status, using self-reported MG symptoms and severity as the input variables.

MG symptom	*p*-value	Odds ratio
Drooping eyelids	**0.0123**	**0.75**
Shortness of breath	**0.0001**	**1.68**
Difficulty chewing	0.3511	0.82
Leg weakness	**0.0013**	**0.67**
Arm weakness	0.4531	1.11
Blurred or double vision	**0.0012**	**1.39**
Difficulty swallowing	**2.81e-12**	**2.67**
Impaired speech	**0.0002**	**2.44**

Independent variables with statistically significant correlation (value of *p* <0.05) with exacerbation status are shown in bold. Odds ratios were calculated from the Beta coefficient for each symptom. Odds ratios demonstrating a positive association with self-reported exacerbations are shown in blue, while those with a negative association are shown in red.

### Association between medication groups and exacerbation profiles

A Fisher-exact test was done to examine observed vs. expected distribution of participants with and without exacerbations between medication groups. This test resulted in a value of *p* of 0.0393, indicating a discrepancy in the observed vs. expected distribution of participants. In our study, group 4 (patients reporting treatments typically reserved for refractory MG), is strongly positively associated with reporting exacerbations, while group 0 (no reported active medications for MG) and 3 (patients reporting treatment with steroid-sparing, chronic immunosuppression) were negatively associated with occurrence of exacerbations (Supplementary Table S2). No significant discrepancy was found between medication groups in self-reported MG-ADL scores and symptoms (data not shown).

### Association between diagnosis groups and exacerbation profiles

Associations between diagnosis groups (classified according to self-reported time since initial diagnosis of MG) and exacerbation profiles were explored. Although the majority (53%) of participants who reported exacerbations were in the recent diagnosis group (within 0–2 years of study enrollment) (Supplementary Table S3), no statistically significant discrepancy in the distribution of participants who reported exacerbations (or who did not) was observed across diagnosis groups (Fisher-exact test value of *p* = 0.311).

However, when a similar analysis was used to examine the total number of days reported as exacerbations or non-exacerbations for participant who reported exacerbations (*N* = 45), a strong positive association was observed between participants in the recent diagnosis group, and the total number of exacerbation days reported (O/E ratio 1.40, see Supplementary Table S4). Moreover, medium and long-term diagnosis groups showed progressive negative association between the time since initial diagnosis, and the total number of exacerbation days reported during the study. A Pearson Chi-square test resulted in a value of *p* <2.2e-16, showing a significant discrepancy in the distribution of reported exacerbation days between diagnosis groups.

When examining the MG-ADL scores of each group, no statistically significant differences between the MG-ADL scores reported by each diagnosis group were observed: Kruskal–Wallis value of *p*s of 0.9559 and 0.2648 for mean MG-ADL scores and maximum MG-ADL scores, respectively (data not shown). This suggests that for this study cohort, participant-reported symptom severity does not vary significantly based on the time from initial diagnosis.

### Symptom clustering during self-reported exacerbations

**Figure 7:**
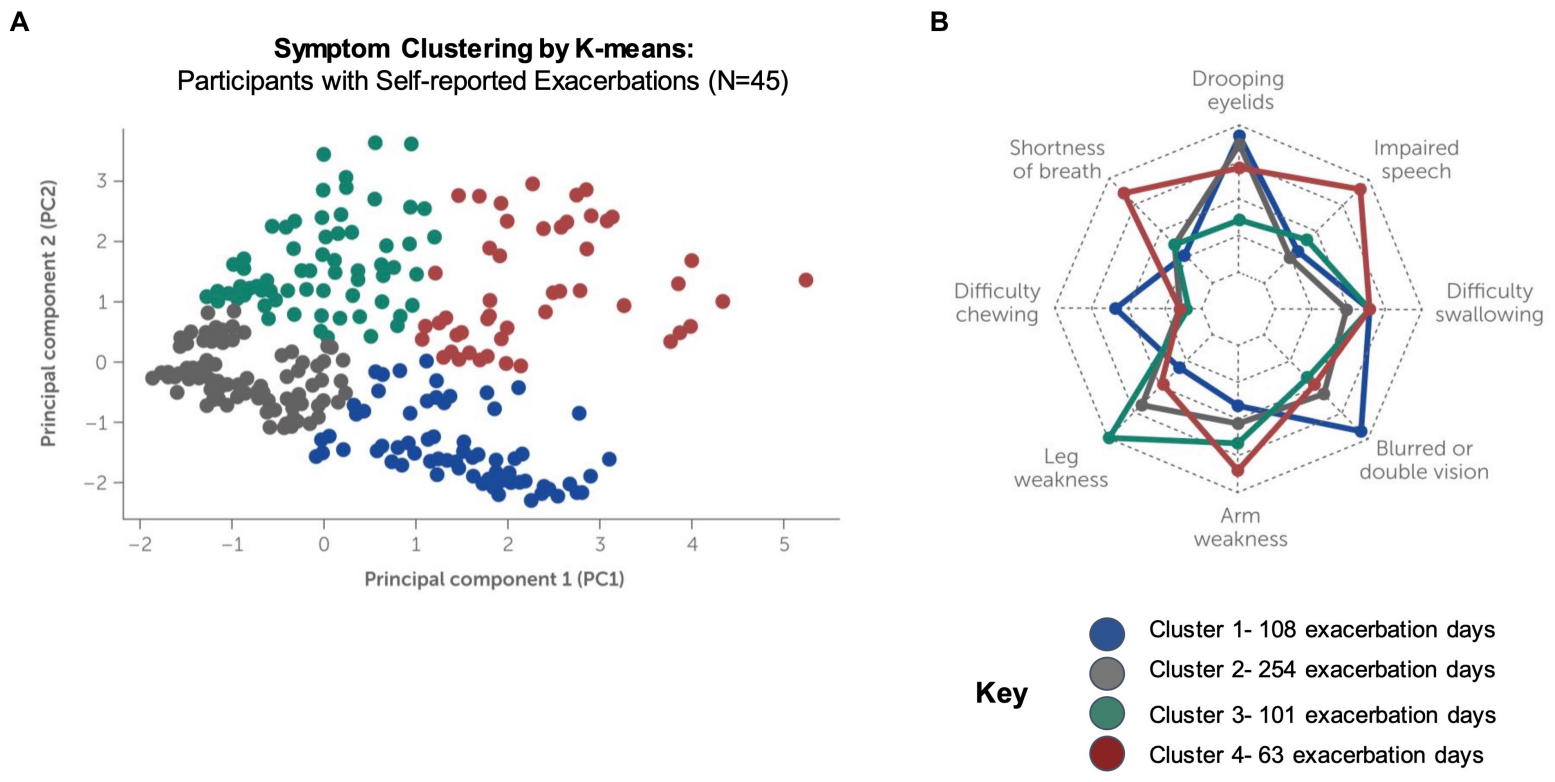
Clustering of symptom signatures for participants reporting exacerbations. Clustering by K-means was conducted using symptom frequency and severity data for participants who self-reported exacerbations during the study (*N* = 4, 525 exacerbation days reported). **(A)** Four distinct clusters were identified, each with a unique signature of symptom occurrence and severity (see Supplementary Figures S1–S4). **(B)** Feature importance analysis (radar plot) for machine-learning driven classification of exacerbations into each cluster.

Unsupervised machine learning methods were applied to the data of participants who reported exacerbations (*N* = 45) to examine symptom signatures and clustering during exacerbation and non-exacerbation days. Using a combination of principal component analysis (PCA) with two components and clustering by K-means, 4 symptom clusters were identified (Figure 7A). The variance contribution of the two principal components and the resulting component matrix of the PCA are shown in Supplementary Tables S5, S6, respectively. A subsequent feature analysis using a one-versus-all random forest classifier was performed to evaluate the combination of symptoms and symptom severity distinguishing each unique cluster from the other clusters, summarized below:

- **Cluster 1**: This cluster demonstrates the highest symptom severity for drooping eyelids and blurred or double vision and is also characterized by the absence of arm and leg weakness.
- **Cluster 2**: Difficulty swallowing, shortness of breath and blurred or double vision are the dominant symptoms in this cluster, with lowest observed severity for all other symptoms.
- **Cluster 3**: This cluster demonstrates increased symptom severity for arm and leg weakness as compared to other clusters, accompanied by drooping eyelids, shortness of breath and difficulty swallowing at lower severities.
- **Cluster 4**: This cluster demonstrates the highest symptom severities for arm and leg weakness, drooping eyelids, shortness of breath and impaired speech as compared to other clusters.

Supplementary Figures S1–S4 summarize the severity of symptoms in each unique cluster and show the order of feature importance for classification into the cluster. The radar plot in Figure 7B summarizes the feature importance for classification of exacerbations into all clusters. Note that feature importance assigns a score to the input variables to determine how important they are for determining the target variable (i.e., the predicted cluster). Feature importance does not correlate with symptom severity, which is shown in for each cluster in Supplementary Figures S1–S4.

## Discussion

This study highlights several interesting patterns of MG symptoms over time in a real-world population of patients. In prior studies, MG-ADL scores of 0 or 1 have been considered consistent with minimal symptom expression (MSE), while MG-ADL scores of 6 or greater have been used to identify patients with high disease burden (5, 17). In addition, the cutoff for a patient acceptable symptom state (PASS) in MG has been previously reported at MG-ADL score of 2 (18). For this study population, the median average MG-ADL score during self-reported exacerbations was 7 (interquartile range 4–9, range 0–19), suggesting moderate to severe disease burden (Figure 5A). Furthermore, for participants who reported experiencing exacerbations during the study period, average MG-ADL scores were significantly higher during periods of symptom exacerbation, as compared to non-exacerbation, indicating concordance between patient-perceived symptom worsening and the clinically accepted definition of a symptom exacerbation (Figure 5B). Patients had the opportunity to select “yes,” “no” or “unsure” as their self-reported exacerbation status in the daily check-in. The fact that 73% of participants together reported a total of 630 days as “unsure” highlights the variable nature of the condition, and how difficult it can be to clearly define an exacerbation event. Added to this, MG patients appear to have differential “baseline” levels of symptom severity, and this may influence their perception of a defined period of symptom worsening. Also, some patients report a period of “not sure” daily check-ins before a self-reported exacerbation, suggesting that there may be a pre-exacerbation period in these patients, which is the subject of future study.

Our data suggest that participant-generated daily step counts may be a useful feature to differentiate between exacerbation and non-exacerbation periods for MG patients (Figure 6). For the subset of participants who contributed step count data from their wearable devices, we observed a weak negative correlation between daily step count and MG-ADL scores. This finding is consistent with prior research demonstrating a reduction in daily steps counts for patients with neurologic disease, as compared to healthy controls (19). However, it should be noted that for a very small subset of participants, physical activity as measured by step count appeared to increase on days reported as exacerbations. It is possible that these participants had been highly active earlier in the day, prior to completing their daily check in, and then reported an exacerbation due to fatigue or worsening symptoms after exertion. Our study did not specifically evaluate levels of fatigue, which is known to be a key dimension of MG symptoms, and thus further work is required to understand the relationship between physical activity, fatigue and clinically significant exacerbations.

Participant-reported symptom representation in the daily check-ins suggests that some symptoms are reported more often than would occur by chance during a self-reported exacerbation. For example, our analyses suggest that difficulty swallowing, and impaired speech are positively associated with exacerbations (i.e., these symptoms are more often reported during periods of exacerbation) (Table 3). Furthermore, drooping eyelids, arm and leg weakness exhibit negative association (i.e., they were more often reported as symptoms during non-exacerbation days). A generalized linear mixed model trained on a subset of participants who reported exacerbations and had high adherence to daily symptom reporting identified impaired speech, difficulty swallowing and shortness of breath as highly predictive variables for determining exacerbation status (Table 4). Taken together, these patterns are likely to be the result of patient perceptions of severity – shortness of breath, difficulty swallowing, and impaired speech could be the precursors of a myasthenic crisis, and therefore these symptoms may be weighed more heavily in patients’ minds when making their determination of worsening symptoms. In contrast, drooping eyelids and extremity weakness may be symptoms more commonly experienced on a “day-to-day” basis, and therefore may be considered by patients as part of their “baseline” status during non-exacerbation days.

It should be noted that this observational study includes patients who were using MG and non-MG indicated medications and did not exclude entry into the study based on concomitant comorbid conditions. We have demonstrated that patients belonging to different medication groups showed a differential likelihood of reporting an exacerbation in symptoms during our study (Supplementary Table S2). Notably, participants in Group 3 (chronic, steroid-sparing immunosuppression) were less likely to experience exacerbations during the study period. In contrast, classification into Group 4 (treatment for refractory disease) was strongly positively associated with reporting exacerbations during the study. One possible interpretation of these results is that some participants reach optimized medical management on chronic immunosuppression (they experience fewer symptom exacerbations), while others continue to experience frequent exacerbations and high symptom burden, requiring treatment for refractory disease. Indeed, a recent retrospective analysis of newly diagnosed generalized MG patients found that 19% of patients remained symptomatic for 2 years after disease onset despite being treated with immunosuppressive therapy, and these patients were more likely to be treatment resistant in the following years (20). Similarly, a strong positive association was observed between participants in the recent diagnosis group (within 0–2 years of study enrollment), and the total number of exacerbation days reported (O/E ratio 1.40, see Supplementary Table S4). Notably, other studies of therapeutic efficacy in MG have excluded patients who were less than 2 years from an MG diagnosis, in order to minimize the risk that a participant with refractory MG would be misclassified as having nonrefractory disease due to insufficient time on treatment to experience no response or inadequate response (5). Our observations support this practice by demonstrating a progressive negative association between medium (2–5 years) and long-term (>5 years) diagnoses and the number of exacerbation days reported during the study.

Unsupervised learning methods applied to the study data determined that some combinations of MG symptoms occur together more often than would be expected by chance. This analysis provides a unique look into the lived experience of these patients during exacerbation periods and demonstrates the potential for this type of digital phenotyping with high-frequency data reporting to uncover patterns in the presentation of the disease across populations of patients. Prior work has classified MG patients into subgroups according to their clinical characteristics at presentation, such as early or late onset disease, serum antibody status and antibody subtype, or the presence or absence of a thymoma (11, 20, 21). To our knowledge, this is the first report identifying homogenous subgroups of MG exacerbations based on symptom signatures. These clusters may be used for hypothesis generation and further study.

There are a couple of important remarks that come with this study. The study may be limited by the representativeness of the study population, and thus the generalizability of the findings to the broader MG patient population. For instance, the decentralized format of the study and digital methods for recruitment, enrollment and data reporting are likely to have been selective for participants with a moderate-to-high level of digital literacy. Furthermore, while the study population was appropriately diverse with regards to age, gender and geographic distribution, there was significant room for improvement on recruiting and retaining ethnically diverse participants. Finally, participants who requested to be withdrawn from the study did so because they felt they were too ill or functionally impaired to continue participating, which may represent a source of attrition bias. Unfortunately, there were several participants who requested to be withdrawn from the study after an acute worsening of MG symptoms following COVID-19 infection, some requiring hospitalization. Though reportedly rare, this is consistent with case reports of MG exacerbation or myasthenic crisis following COVID-19 infection (22–24).

Overall, our study suggests that decentralized, smartphone-based methods to collect real-world data from MG patients are feasible and may provide enhanced visibility into the lived experience of MG patients. Furthermore, the results summarized above suggest that some participant-reported data streams may be useful as features for development of a composite model to predict oncoming exacerbations in MG patients. These features include dynamic changes in day-to-day symptom reporting and severity, daily step counts as a measure of physical activity, and clinical characteristics of the patient, including the amount of time since their initial diagnosis and their active medications related to MG treatment. Finally, by applying unsupervised machine learning methods, we were able to identify unique clusters of exacerbation subtypes, each with their own specific representation of symptoms and symptom severity. While these symptom signatures require further study and validation, our results suggest that digital phenotyping, characterized by increased multidimensionality and frequency of the data collection, holds promise for furthering our understanding of clinically significant exacerbations and reimagining the approach to treating MG as a “snowflake” condition.

## Data availability statement

The raw data supporting the conclusions of this article will be made available by the authors, without undue reservation.

## Ethics statement

The studies involving human participants were reviewed and approved by Salus IRB. The patients/participants provided their written informed consent to participate in this study.

## Author contributions

SS, FR, and HD contributed to the data analysis, manuscript conceptualization, drafting, and revision. EL, J-CS, CS, ML, and ZF contributed to the manuscript revisions. All authors contributed to the article and approved the submitted version.

## Funding

This study received funding from UCB Pharma. The funder was not involved in the study design, collection, analysis or interpretation of data—these were undertaken by Sharecare Inc., as the study sponsor. UCB Pharma was involved in the decision to publish, and the preparation of this manuscript. Medical writing support was paid for by UCB Pharma.

## Conflict of interest

EJ and J-CS are employees and stockholders of UCB Pharma. HD is a stockholder and former employee of UCB Pharma. SS, ZF, and ML are stockholders and former employees of Sharecare, Inc. CS and FR are employees and stockholders of Sharecare, Inc. This study received funding from UCB Pharma.”

## Publisher’s note

All claims expressed in this article are solely those of the authors and do not necessarily represent those of their affiliated organizations, or those of the publisher, the editors and the reviewers. Any product that may be evaluated in this article, or claim that may be made by its manufacturer, is not guaranteed or endorsed by the publisher.
