# Supplementary material for: A decentralized, prospective, observational study to collect real-world data from patients with myasthenia gravis using smartphones
Source: Front Neurol. 2023 Aug 1;14:1144183. doi: 10.3389/fneur.2023.1144183 (PMC10427188; doi:10.3389/fneur.2023.1144183)
Supplement: Supplementary file 1 [file Data_Sheet_1.docx]

# Supplementary Material

# Supplementary Figures

**Supplementary Figure 1: Results of symptom clustering during self-reported exacerbations.** (A) Symptom occurrence and severity signature for cluster 1. (B) Visualization of feature importance to classify a patient into cluster 1.


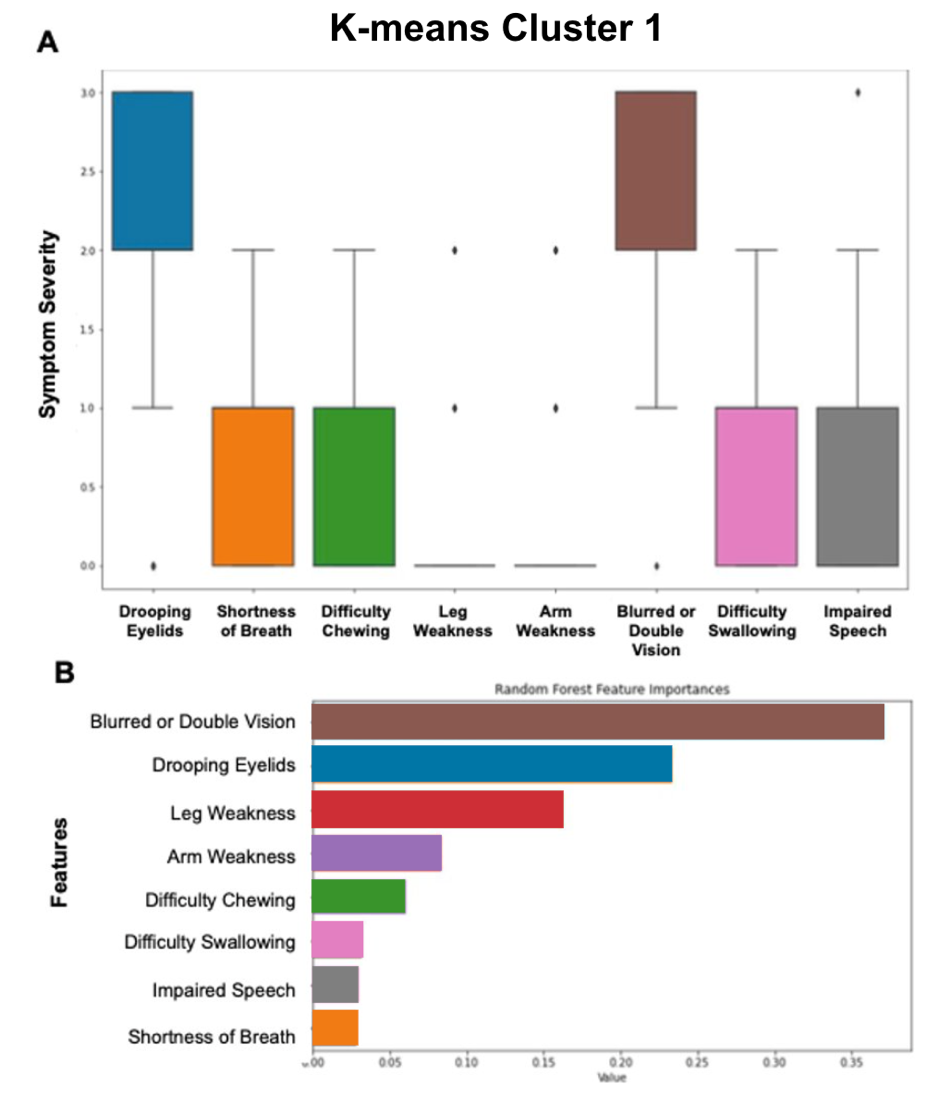


**Supplementary Figure 2: Results of symptom clustering during self-reported exacerbations.** (A) Symptom occurrence and severity signature for cluster 2. (B) Visualization of feature importance to classify a patient into cluster 2.


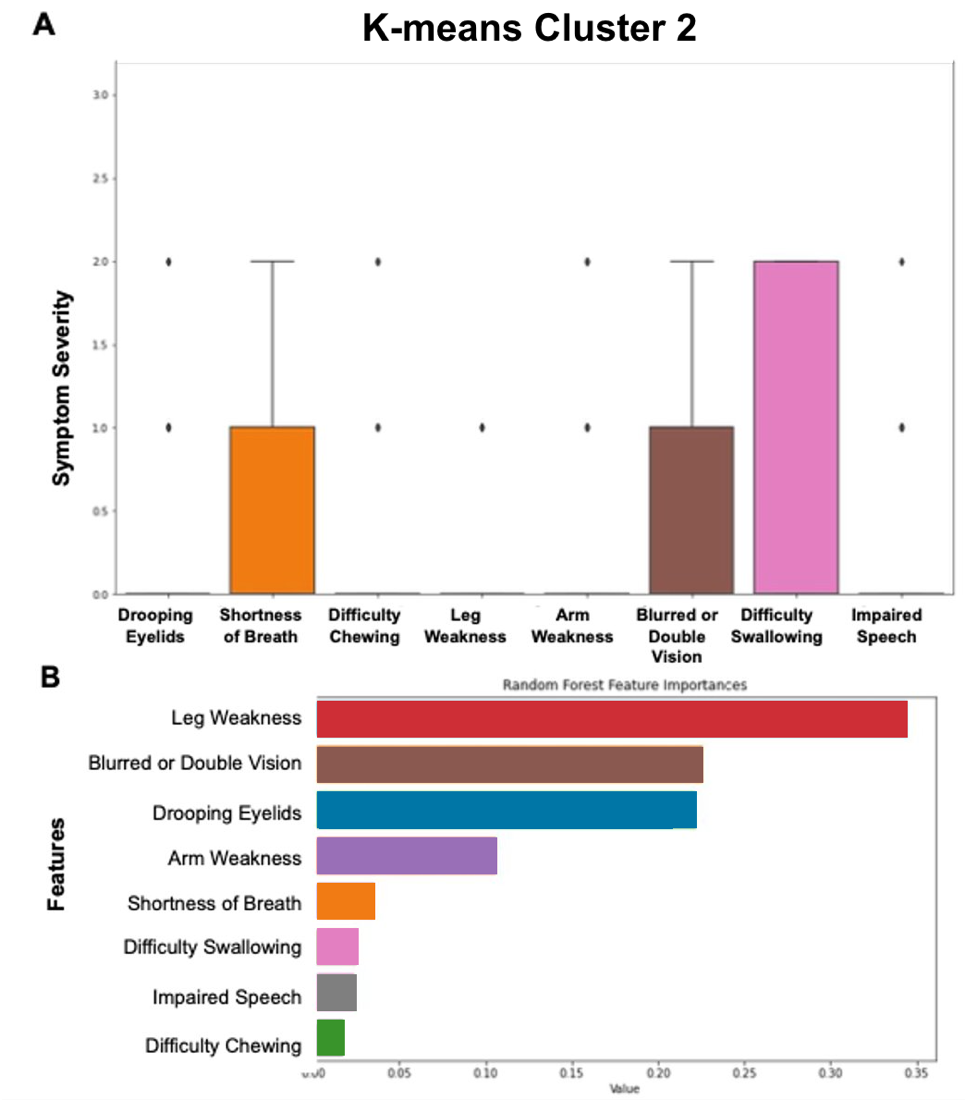


**Supplementary Figure 3: Results of symptom clustering during self-reported exacerbations.** (A) Symptom occurrence and severity signature for cluster 3. (B) Visualization of feature importance to classify a patient into cluster 3.


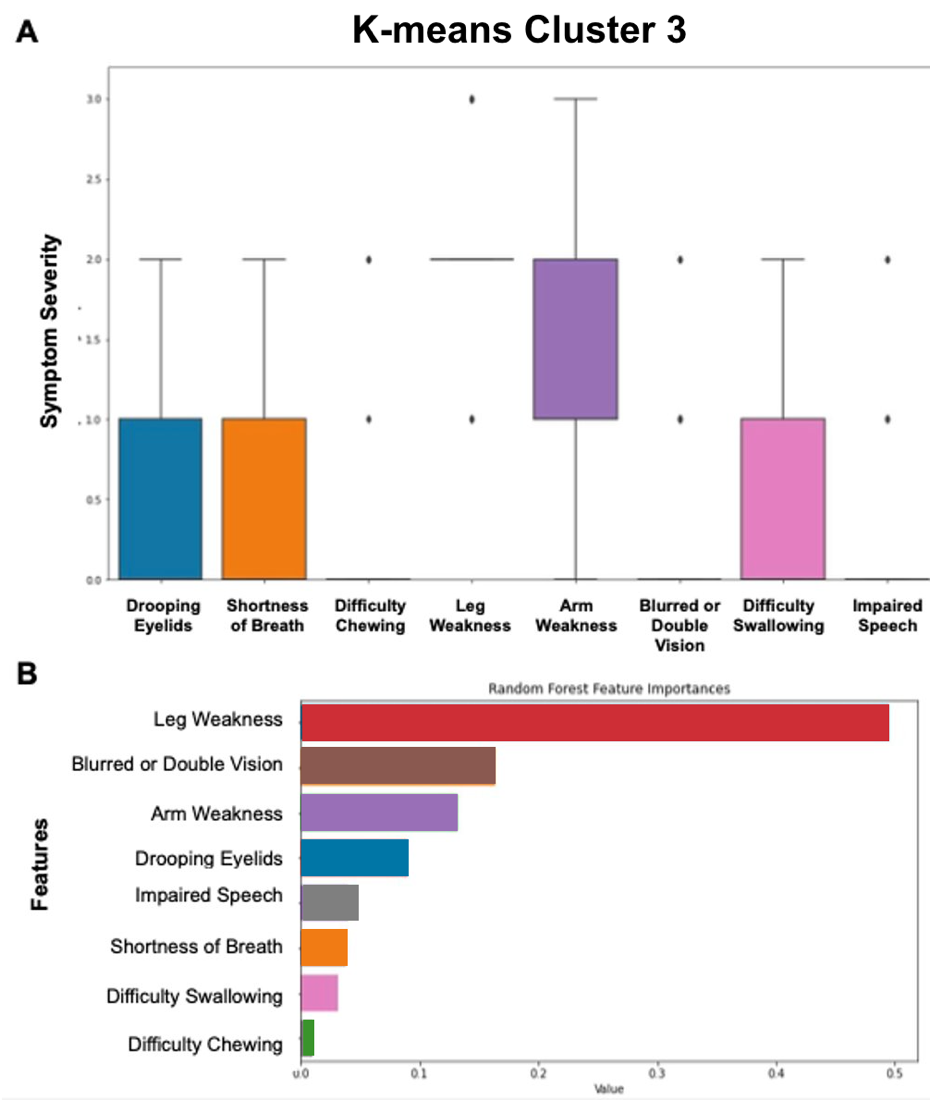


**Supplementary Figure 4: Results of symptom clustering during self-reported exacerbations.** (A) Symptom occurrence and severity signature for cluster 4. (B) Visualization of feature importance to classify a patient into cluster 4.


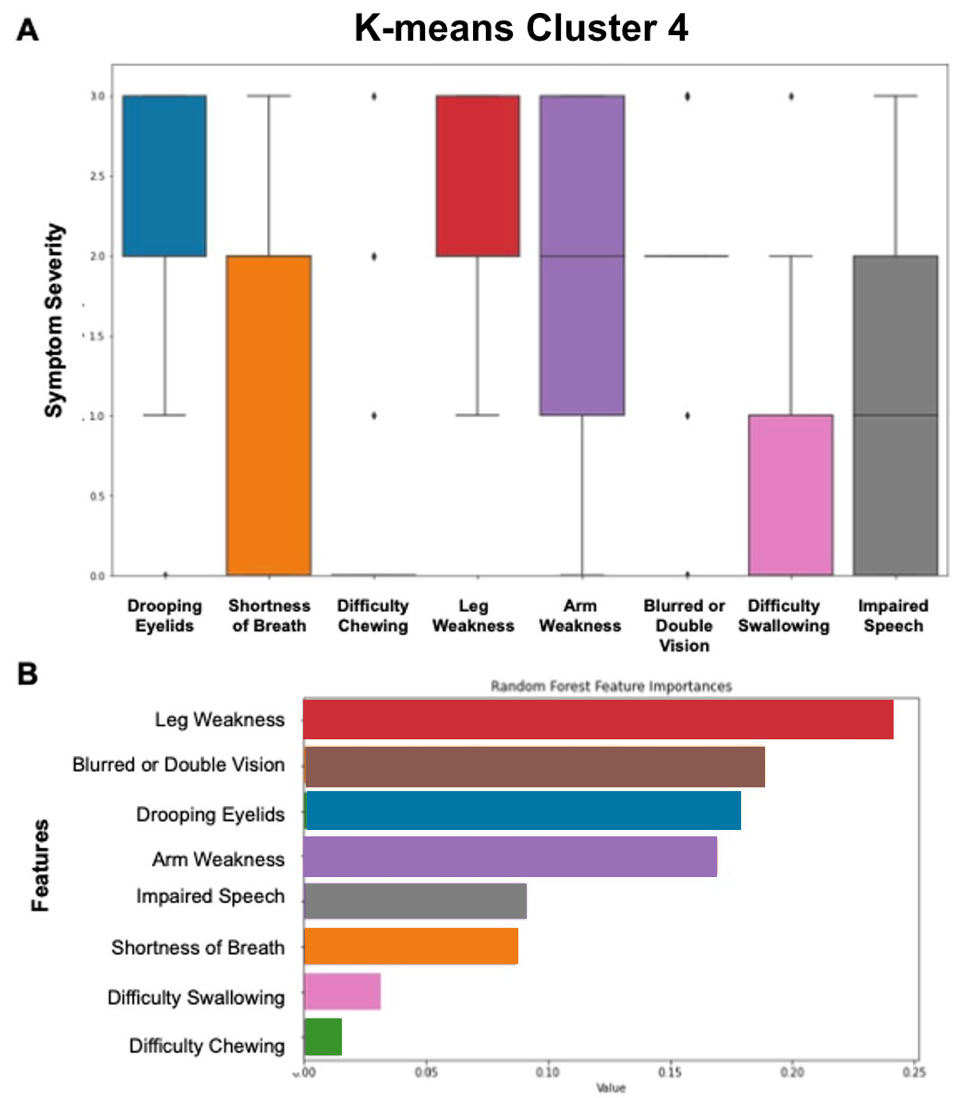


# Supplementary Tables

**Supplementary Table 1**: Expected Number of Symptom Exacerbations During a 3-month period, by Target Enrollment. The average number of expected exacerbations per year across all MG patients is approximately 3 (unpublished data). Using this number, we explored the average probability of capturing a symptom exacerbation in the study time frame. We expected to capture 75 flares across 3 months if the sample size was set to 100 patients, with a minimum of 65 and maximum of 85.

|  | **Minimum** | **Target** | **Maximum** |
| --- | --- | --- | --- |
| **Participants** | 50 | 100 | 200 |
| **Length of Study (in years)** | 0.25 | 0.25 | 0.25 |
| **Average Number of Exacerbations, Per Patient, Per Year*** | 3 | 3 | 3 |
| **Expected Number of Exacerbations within Study Timeframe** | 37.5 | 75 | 150 |

**Supplementary Table 2**: Schedule of assessment for electronic participant-reported outcomes (ePROs) and participant-generated health data streams collected during the study.

| **Participant-reported Outcomes Data** | | | |
| --- | --- | --- | --- |
| **Study Phase** | **Instrument and Frequency** | **Data Points** | **Description** |
| **Onboarding** | **MG Initial Survey**  **(Once)** | Age | Age at the time of study enrollment |
|  |  | Gender | Sex assigned at birth (Male/Female) |
|  |  | Race/Ethnicity | Participants selected from these possible choices (could select more than one):  -White  -Black or African American  -Hispanic, LatinX or Spanish origin  -American Indian or Alaska Native  -Asian  -Other |
|  |  | Location | Location (lat/long) coordinates at start of study |
|  |  | Disease duration | Age at study start - age at MG diagnosis |
|  |  | Symptom Frequency | How often do you have an exacerbation?  -Monthly  -Quarterly  -Yearly |
|  |  | Time between exacerbations | What is the typical time between exacerbations?  -Weeks  -Months  -Years |
|  |  | Exacerbation Duration | How long do your exacerbations typically last?  -Less than one week  -One week  -Two weeks  -More than two weeks |
|  |  | Active Medications | Participants were asked to select from a dropdown menu of medications, or enter a medication name as free text. |
|  |  | Comorbid Conditions | Participants were asked to select from a dropdown menu of conditions, or enter a condition name as free text. |
|  | **MG-ADL Baseline Survey**  **(Once)** | For GOOD and BAD Moments: Presence and severity of 8 MG symptoms | MG Symptoms rated:   - - Vocal hoarseness (dysarthria)   - Chewing   - Swallowing (dysphagia)   - Breathing (dyspnea)   - Arm motor weakness   - Leg motor weakness   - Blurred vision   - Eyelid droop (ptosis)   Symptoms rated on a severity scale of 0-3  A GOOD Day and BAD Day MG-ADL score are generated (0-24) from the baseline survey. |
| **Study Course** | **Daily Check in**  (Daily- 90 possible check ins total for the study course) | How do you feel today? | OK/Not OK selected. If Not OK was selected, Participant was asked to select from 8 symptoms in the MG-ADL instrument and then rate the severity of selected symptoms |
|  |  | MG Symptoms and severity (MG-ADL Assessment) | MG Symptoms for selection and rating (0-3 severity scale) rated:   - - Vocal hoarseness (dysarthria)   - Chewing   - Swallowing (dysphagia)   - Breathing (dyspnea)   - Arm motor weakness   - Leg motor weakness   - Blurred vision   - Eyelid droop (ptosis) |
|  |  | Alleviating, Exacerbating factors | Are there any factors that have changed since yesterday?  Participants could select “No changes since yesterday” OR select from:  -Change in medication  -Diet  -Exercise  -Pain  -General fatigue  -Muscle fatigue  -Sleep  -Mood  -Stress  -Temperature/weather  -Menstruation  Once selected- factors could be rated as contributing on the following scale:  -Less than normal  -Normal  -More than normal  -Not sure |
|  |  | Exacerbation Status | Do you believe an exacerbation is coming on, or in progress?  Participants could select:  -Yes  -No  -Not Sure |
| **Participant-generated Health Data** | | | |
| **Study Phase** | **Data Point** | **Description** | |
| **Study Course** | **Daily Step Count** | Participants could make optional connections for Activity Data, Daily Step Count.  Integrated data collection occurred via Apple Health, Google Fit or Validic integrations with the associated participant permissions. | |

**Supplementary Table 3:** Classification of study participants into medication groups, by exacerbation status (N= 75). Observed/expected (O/E) ratios are indicated between parentheses. Cells marked in red show values that are less than what would be expected to occur by chance, whereas cells marked in blue show values that are higher than would be expected to occur by chance.

| **Medication Group** | **Participants without Exacerbations**  **(O/E ratio)** | **Participants with Exacerbation**  **(O/E ratio)** |
| --- | --- | --- |
| **Group 0**  **(No Reported Medications) (n=16)** | 9 **(1.41)** | 7 **(0.73)** |
| **Group 1** **(Symptomatic Therapy) (n=15)** | 5 (0.83) | 10 (1.11) |
| **Group 2 (Pyridostigmine + Glucocorticoids) (n=9)** | 4 (1.11) | 5 (0.93) |
| **Group 3** **(Steroid-sparing Chronic Immunosuppression) (n=13)** | 8 **(1.54)** | 5 **(0.64)** |
| **Group 4** (**Treatment for Severe, Refractory MG) (n=22)** | 4 **(0.45)** | 18 **(1.37)** |

**Supplementary Table 4:** Classification of study participants into diagnosis groups, by exacerbation status (N=73, 2 participants did not report time of initial diagnosis). Observed/expected (O/E) ratios are indicated between parentheses.

| **Diagnosis Group** | **Participants with Exacerbations**  **(O/E ratio)** | **Participants without Exacerbations**  **(O/E ratio)** |
| --- | --- | --- |
| Recent (0-2 years) (n=39) | 24 (1.10) | 15 (0.89) |
| Medium (2-5 years) (n=12) | 5 (0.65) | 7 (1.42) |
| Long (>5 years) (n=22) | 15 (1.05) | 7 (0.94) |

**Supplementary Table 5:** Total study days with and without self-reported exacerbations for participants who reported exacerbations, by diagnosis group (N=44, 1 participant did not report time of initial diagnosis). Observed/expected (O/E) ratios are indicated between parentheses. Cells marked in red show values that are less than what would be expected to occur by chance, whereas cells marked in blue show values that are higher than would be expected to occur by chance.

| **Diagnosis Group** | **Days with Exacerbations** **(O/E ratio)** | **Days without Exacerbations**  **(O/E ratio)** |
| --- | --- | --- |
| Recent (0-2 years) (n=24) | 359 **(1.40)** | 711 (0.87) |
| Medium (2-5 years) (n=5) | 12 **(0.40)** | 112 (1.19) |
| Long (>5 years) (n=15) | 106 **(0.57)** | 687 (1.14) |

**Supplementary Table 6:** Variance contribution of each principal component (PC) from the PCA analysis that was used as input of the K-means clustering.

|  | PC1 | PC2 |
| --- | --- | --- |
| No Exacerbation | 2.76 | 1.19 |
| Exacerbation | 2.49 | 1.14 |

**Supplementary Table 7:** Component matrix resulting from the PCA analysis that was used as input of the K-means clustering.

|  | PC1 | PC2 |
| --- | --- | --- |
| Drooping Eyelids | 0.612 | -0.170 |
| Shortness of Breath | 0.268 | 0.080 |
| Difficulty Chewing | 0.128 | -0.121 |
| Leg Weakness | 0.285 | 0.679 |
| Arm Weakness | 0.288 | 0.536 |
| Blurred or Double Vision | 0.568 | -0.446 |
| Difficulty Swallowing | -0.056 | -0.043 |
| Impaired Speech | 0.215 | 0.008 |
